# Modelling the impact of condition-dependent responses and lipid-store availability on the consequences of disturbance in a cetacean

**DOI:** 10.1093/conphys/coac069

**Published:** 2022-11-17

**Authors:** Alec Burslem, Saana Isojunno, Enrico Pirotta, Patrick J O Miller

**Affiliations:** Sea Mammal Research Unit, Scottish Oceans Institute, School of Biology, University of St Andrews, St Andrews, Fife KY16 8LB, UK; Sea Mammal Research Unit, Scottish Oceans Institute, School of Biology, University of St Andrews, St Andrews, Fife KY16 8LB, UK; Centre for Research into Ecological and Environmental Modelling, School of Mathematics, The Observatory, Buchanan Gardens, University of St Andrews, St Andrews, Fife KY16 9LZ, UK; Centre for Research into Ecological and Environmental Modelling, School of Mathematics, The Observatory, Buchanan Gardens, University of St Andrews, St Andrews, Fife KY16 9LZ, UK; Sea Mammal Research Unit, Scottish Oceans Institute, School of Biology, University of St Andrews, St Andrews, Fife KY16 8LB, UK

**Keywords:** Body condition, population consequences of disturbance, sperm whale

## Abstract

Lipid-store body condition is fundamental to how animals cope with environmental fluctuations, including anthropogenic change. As it provides an energetic buffer, body condition is expected to influence risk-taking strategies, with both positive and negative relationships between body condition and risk-taking posited in the literature. Individuals in good condition may take more risks due to state-dependent safety (‘ability-based’ explanation), or alternatively fewer risks due to asset protection and reduced need to undertake risky foraging (‘needs-based’ explanation). Such state-dependent responses could drive non-linear impacts of anthropogenic activities through feedback between body condition and behavioural disturbance. Here, we present a simple bioenergetic model that explicitly incorporates hypothetical body condition-dependent response strategies for a cetacean, the sperm whale. The model considered the consequences of state-dependent foraging cessation and availability of wax ester (WE) lipids for calf provisioning and female survival. We found strikingly different consequences of disturbance depending on strategy and WE availability scenarios. Compared with the null strategy, where responses to disturbance were independent of body condition, the needs-based strategy mitigated predicted reductions in provisioning by 10%–13%, while the ability-based strategy exaggerated reductions by 63%–113%. Lower WE availability resulted in more extreme outcomes because energy stores were smaller relative to the daily energy balance. In the 0% availability scenario, while the needs-based strategy reduced deaths by 100%, the ability-based strategy increased them by 335% relative to null and by 56% relative to the same strategy under the 5%–6.7% WE availability scenario. These results highlight that state-dependent disturbance responses and energy store availability could substantially impact the population consequences of disturbance. Our ability to set appropriate precautionary disturbance thresholds therefore requires empirical tests of ability- vs needs-based response modification as a function of body condition and a clearer understanding of energy store availability.

## Teaser

Body condition-dependent risk-taking is observed in many animal taxa but is little explored in cetaceans. We used model simulations to explore how uncertainties surrounding this behaviour and the metabolic physiology of deep-diving cetaceans may be limiting our understanding of the energetic consequences of anthropogenic disturbance.

## Introduction

When making behavioural decisions, animals must balance multiple opportunities and threats, including reproductive investment and the risks of predation and starvation (Houston *et al*., 1993; Houston and McNamara, 1999). The risk-disturbance hypothesis posits that behavioural response to anthropogenic disturbance involves the same economic principles as prey decision-making under predation risk and predicts that response decisions depend on the perceived risk relative to the cost–benefit of the response (Frid and Dill, 2002; Beale and Monaghan, 2004a). Since responses specific to anthropogenic disturbance have not had time to evolve in many long-lived species, perceived risk may be driven by generalised features of the disturbance source broadly perceived as threatening, such as approach speed and directness (Frid and Dill, 2002). State-dependent mechanisms that evolved to optimize predation risk trade-offs may therefore also influence responses to anthropogenic sources of disturbance.

Body condition is a state variable that can influence state-dependent behavioural responses (Moran *et al*., 2020). It also represents a potential physiological outcome of those responses, since foraging cessation or energetic costs associated with the behavioural response may lead to a reduction in net energy intake. Moreover, body condition mediates the consequences of behavioural responses on the population through effects on survival and reproductive investment (McNamara and Houston, 1996; Hall *et al*., 2001). If response intensity both drives and is driven by changes in body condition, this may therefore cause non-linear impacts through feedback processes (Sih *et al.*, 2015). Body condition influences risk-taking in a variety of taxa and has been shown also to influence responses to anthropogenic disturbance (Madsen, 1995; Seltmann *et al.*, 2012; Harding *et al*., 2020). Both positive and negative relationships between body condition and risk-taking are posited in the literature (Sih *et al.*, 2015; Moran *et al*., 2020). ‘Needs-based’ explanations, such as the starvation–predation trade-off, propose that, as the amount of stored energy increases, the risk of starvation tends to decrease relative to that of predation. This reduces the need to take risks to obtain foraging rewards (Clark, 1994). In contrast, the state-dependent safety hypothesis posits a positive relationship between body condition and risk-taking, whereby good condition confers enhanced fight or flight abilities to the individual, reducing the expected cost of an encounter with a predator (‘ability-based’ explanation; Luttbeg and Sih, 2010). These strategies are not mutually exclusive and are likely to operate together at different strengths depending on stimuli, environmental and individual context (Luttbeg and Sih, 2010; Sih *et al.*, 2015). In such cases, the net effect in a given context will depend on the relative magnitude of these competing drivers (Sih *et al.*, 2015). For example, an animal in extremely poor condition may take significant predation risks to continue foraging (needs-based behaviour). This may occur even if the average cost of an encounter with a predator is higher than for individuals in better condition (state-dependent safety) if the starvation risk outweighs it. Moran *et al*. (2020) reviewed and reanalysed data from 126 body condition manipulation experiments and found that negative correlations between body condition and risk-taking are more common, consistent with needs-based explanations. Crucially, this effect was observed in response to both predation risk and novel stimuli. However, the authors note that these results do not preclude ability-based strategies to be dominant in certain contexts and the direction of the overall relationship in individual species is likely to depend on their specific defence mechanisms. The risk-disturbance hypothesis predicts that responses to anthropogenic sources of disturbance would depend on these same factors.

Population consequences of disturbance (PCoD) models are important tools for understanding the conservation importance of anthropogenic disturbance, particularly in long-lived, highly mobile marine mammals, for which direct study of vital rates is often impractical (Pirotta *et al*., 2018; Booth *et al*., 2020). PCoD models can help to overcome this problem by simulating, among other pathways, the effects of disturbance on an individual’s energy budget and, in turn, vital rates. Such simulation models rely on a detailed mechanistic understanding of the drivers underpinning disturbance response behaviours and their effects on energetic balance (Pirotta *et al*., 2018; Booth *et al*., 2020). Uncertainties surrounding our understanding of state-dependent response strategies and energy storage physiology may therefore limit our ability to model population-level impacts accurately (Keen *et al*., 2021).

In cetaceans, sensitivity to disturbance is positively correlated with intensity of response to predators, consistent with the risk-disturbance hypothesis (Miller *et al*., 2022). Responses also vary markedly across individuals within species. It is unknown what proportion of this variability reflects dynamic state-dependent processes as opposed to consistent inter-individual differences and stochastic environmental variability (Ellison *et al*., 2012; Harris *et al*., 2018). There are some plausible mechanisms supporting the evolution of an ability-based response strategy in marine mammals. For example, cetaceans that have greater diving capabilities than their predators may utilize depth as a refuge (Aguilar de Soto *et al*., 2020). Depth of neutral buoyancy, oxygen storage and nitrogen loading have all been found to vary with body condition in odontocetes (Miller *et al*., 2004; Lonati *et al*., 2015; Choy *et al*., 2019), which could affect their ability to utilize these refugia. However, few studies have investigated the relationship between body condition and anti-predation or risk-taking behaviour in marine mammals. Beltran *et al*. (2021) found that Northern elephant seals (*Mirounga angustirostris*) under risk of detection by visual predators (white sharks and killer whales) followed a needs-based strategy when deciding to rest or forage. As their condition improved over the course of the season, they sacrificed increasing amounts of the most energy-efficient nocturnal foraging time to rest in the comparative safety of darkness. Siegal *et al.* (2022) found a negative relationship between body condition and anti-predator behaviours in northern bottlenose whales (*Hyperoodon ampullatus*), suggesting a positive relationship between body condition and risk-taking, which corresponds to an ability-based strategy over the range of body conditions observed in that study.

The role of lipids is unusual in marine mammals compared with other mammals, and this too complicates modelling the effects of disturbance in these taxa. In marine mammals, in addition to energy storage, lipids play acoustic, thermal insulation, structural and hydrodynamic roles (Bagge *et al*., 2012; Koopman, 2018). The extent to which lipids are used as an easily accessed energy store versus other functions is thought to vary across marine mammal species depending on habitat, life history strategy and diving behaviour (Koopman, 2007, 2018; Bagge *et al.*, 2012; Kershaw *et al.*, 2019), but is not well understood. This uncertainty is particularly apparent in deep-diving odontocetes. All odontocetes have high proportions of wax ester (WE) lipids in their acoustic apparatus, but deep divers such as sperm whales (*Physeter macrocephalus*) and beaked whales (*Ziphiidae*) also have high concentrations of WEs in their blubber (up to ~90%; Koopman, 2007, 2018). WEs are more buoyant and more thermally insulating than triacylglycerides (TAGs), and these features have been hypothesised to potentially assist in deep diving. However, it is not clear to what extent a blubber rich in WEs represents an adaptation to deep diving or a shared phylogenetic lineage between *Physeteridae* and *Ziphiidae* (Koopman, 2018). WEs are also more energy dense than TAGs but are hydrolysed at only about one tenth their efficiency in most mammals (Place, 1992; Koopman, 2007, 2018). Since WEs are present at such high concentrations in the blubber of sperm whales, the degree of their bioavailability via catabolism may translate into a large difference in resilience to foraging interruption. Kershaw *et al.* (2019) proposed that deep-diving beaked whales may not mobilize large proportions of their blubber lipids even in response to severe nutritional stress. Farmer *et al.* (2018) estimated, through simulation, that storing energy as a combination of WEs and TAGs may reduce sperm whales’ resilience to feeding disruption by shortening their survival time by ~30%, relative to storage as pure TAGs.

Here, we present a bioenergetic simulation model that explicitly incorporates body condition-dependent disturbance responses. We use model simulations to explore the consequences of alternative, hypothetical body condition-dependent strategies on survival and reproductive investment for nursing female sperm whales, under diverse WE availability and disturbance scenarios. While our model is parameterized for sperm whales, the conclusions are likely to be generally applicable to long-lived mammals for which provisioning is a major cost of reproduction.

## Materials and methods

### Bioenergetic model

**Figure 1:**
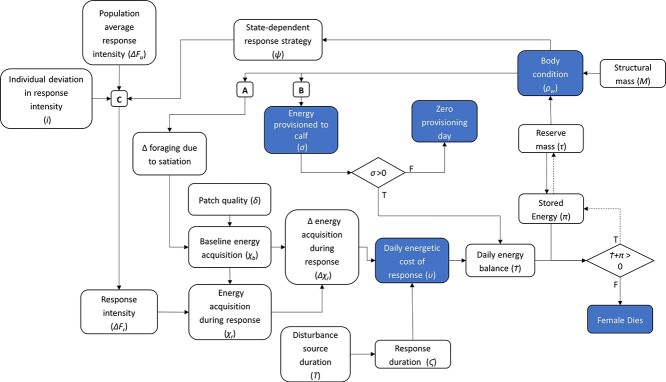
A mechanistic framework for modelling the energetic consequences of state-dependent behavioural response to disturbance. This figure represents the key calculations performed during one daily iteration of the model. Lettered boxes indicate key transfer functions shown in Fig. 2. Diamonds show logic gates for Boolean variables. Boxes in blue are outcome variables reported in the Results section. Dashed lines indicate transfer to the next day in the simulation.

**Figure 2:**
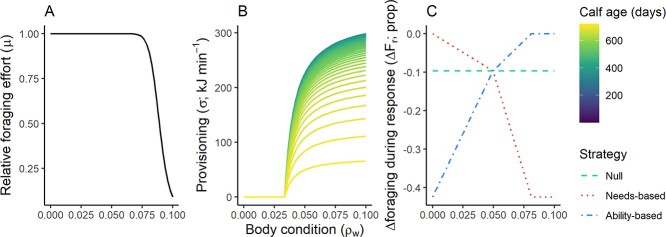
Graphical representations of the lettered transfer functions shown in Fig. 1. (A) Relative foraging effort declines rapidly with body condition above the target level (ρ_t_; see ‘Resource acquisition’). (B) Provisioning of milk to the calf decreases exponentially with increasing calf age and reducing maternal condition, with maternal lactation threshold (ρ_l_) and target body condition (ρ_t_) controlling the occurrence and degree of provisioning (see ‘Provisioning to offspring’). (C) Changes in foraging intensity during response (Δ*F_r_*) as a function of body condition, under three different response strategies. Responses were either independent of body condition (null strategy) or state-dependent, whereby risk-taking either increased or decreased with body condition (ability- or needs-based strategy, respectively; see ‘Behavioural response to disturbance’). Thresholds (ρ_t_ and ρ_l_) vary between WE availability scenarios (see Table 2 and ‘Lipid-store body condition’); for simplicity, the relationships shown here are for the WE-06 scenario and stochastic variability is not shown.

The purpose of this simulation model is to understand how state-dependent response strategies and WE availability may influence the effect of disturbance on nursing female sperm whales. The model contains parameters that can vary between individuals, by behavioural response strategy, by WE availability and over time. Responses to disturbance were specified according to three alternative state-dependent strategies described below and in Table 2 and Fig. 2C. Individuals within each strategy group can vary by body length and starting body condition. Individual body condition was then updated daily, and state-dependent behaviours were modified accordingly. Our simulation model covers the lactation and post-breeding recovery periods of physically mature females, i.e. the interbirth interval minus the duration of pregnancy (Chiquet *et al*., 2013). See Fig. 1 for an overview of the daily model calculations.

Our approach to modelling body condition-dependent behaviours required setting body condition thresholds (see ‘Lipid-store body condition’ and Fig. 2). Such thresholds must be appropriate for simulated populations but are not available empirically (Hin *et al.*, 2019). We therefore followed a two-stage approach. First we simulated body condition on Day 1 only, using the starting lipid reserve values (*ρ_w_* and *τ,* respectively; Table 2, line 22–23, where *d* = 1), of 10^6^ animals for each WE availability scenario (see ‘Scenarios of WE availability’ and Table 1). We then used the results of these ‘initial’ body condition simulations to set the threshold values (maternal lactation threshold, *ρ_l_*; target body condition, *ρ_t_*; and central body condition, *ρ_r_*) used in ‘full’ model simulations (see ‘Lipid-store body condition’ and Table 1). Full model simulations proceeded through time, performing the full set of daily model calculations described in Fig. 1 and Table 2, and were run for all WE scenarios under baseline and disturbed conditions (see ‘Simulation of exposure to disturbance’ and Table 3).

**Table 1:** WE availability scenarios

WE scenario	***λ***_***w***_	***ρ***_***l***_	***ρ***_***r***_	***ρ***_***t***_
WE-00	${\lambda}_w=0$	0.0253	0.0415	0.0723
WE-06	${\lambda}_w\sim Uniform\ (\mathrm{0.05,0.067})$	0.0335	0.0495	0.0809
WE-40	${\lambda}_w\sim Uniform(\mathrm{0.30,0.50})$	0.0693	0.0964	0.1416

WE availability values (λ_w_) varied between scenarios, indicating that WEs were completely unavailable (WE-00), partially available (WE-06) or highly available (WE-40; see ‘Scenarios of WE availability’). Critical body condition thresholds were set based on initial simulations of starting body condition for each scenario (ρ_l_, body condition threshold of lactation; ρ_t_, target body condition, affecting foraging effort; ρ_r_, mean population body condition). For more details, see ‘Lipid-store body condition’

**Table 2:** Overview of model variables and parameters

	Variable/parameter (name in R code, where different from notation)	Notation	Value	Unit	Refs.
	Simulation				
1	Day index (d)	$d$	$1:1004$	Days	Chiquet *et al.* (2013), Farmer *et al.* (2018)
2	Individual index	$w$	$1:3000$		
3	Disturbance duration (dis_dur)	$T(d)$	*See scenarios* *Table 3*.	Min day^−1^	
	Body size				
4	Length (L)	$L(w)$	$L\sim Uniform(\mathit{\min}=10.9,\mathit{\max}=12)$	m	Lockyer (1976), McClain *et al*. (2015)
5	Mass (M)	$M(w)$	$M=1.10\cdot 19.6\cdot (L{(w)}^{2.74})$	kg	Lockyer (1991)
	Lipid stores				
6	Blubber as proportion of total mass (prop_blub)	${P}_b(w)$	${P}_b(w)\sim Uniform(\mathit{\min}=0.31,\mathit{\max}=0.32)$	Prop.	Lockyer (1991)
7	Lipids as proportion of blubber (lip_blub)	${\varLambda}_b(w)$	${\varLambda}_b(w)\sim Beta( shape1=9.812, shape2=13.661)$ *Shape parameters estimated from mean = 0.418, SD = 0.0997*	Prop.	Evans *et al*. (2003)
8	WEs as a proportion of blubber lipid (prop_WE_blub)	${\omega}_b(w)$	${\omega}_b(w)\sim Uniform(\mathit{\min}=0.613,\mathit{\max}=0.990)$	Prop.	Lockyer (1991), Koopman (2007)
9	TAGs as a proportion of blubber lipid (prop_TAG_blub)	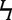 $_b(w)$	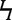 $_b=1-{\omega}_b(w)$	Prop.	
10	Viscera as a proportion of total mass (prop_vis)	${P}_v$	${P}_v=0.09$	Prop.	Lockyer (1991)
11	Lipids as a proportion of viscera (lip_vis)	${\varLambda}_v(w)$	${\varLambda}_v(w)\sim Uniform(\mathit{\min}=0.6944,\mathit{\max}=0.8043)$	Prop.	Lockyer (1991)
12	WE proportion of visceral lipids (prop_WE_vis)	${\omega}_v$	${\omega}_v=0.4508$	Prop.	Lockyer (1991), Farmer *et al*. (2018)
13	TAGs as a proportion of visceral lipids (prop_TAG_vis)	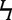 $_v$	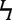 $_v=1-{\omega}_v$	Prop.	
14	Muscle as a proportion of total mass (prop_musc)	${P}_m(w)$	${P}_m(w)\sim Uniform(\mathit{\min}=0.225,\mathit{\max}=0.3)$	Prop.	Lockyer (1991)
15	Lipids as proportion of muscle (lip_musc)	${\varLambda}_m(w)$	${\varLambda}_m\sim Beta( shape1=0.660, shape2=22.254)$ *Shape parameters estimated from mean = 0.0288, SD = 0.0342*	Prop.	Lockyer (1991), Farmer *et al*. (2018)
16	WEs as a proportion of muscle lipid (prop_WE_musc)	${\omega}_m$	${\omega}_m=0.154$	Prop.	Lockyer (1991)
17	TAGs as a proportion of muscle lipid (prop_TAG_musc)	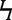 $_m$	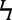 $_m=1-{\omega}_m$	Prop.	
18	Total body WE mass (WE_tot)	${\alpha}_w(w)$	${\alpha}_w(w)$ $=M(w)\cdot {P}_b(w)\cdot {\varLambda}_b(w)\cdot {\omega}_b(w)+M(w)\cdot {P}_v\cdot {\varLambda}_v(w)\cdot {\omega}_v+M(w)\cdot {P}_m(w)\cdot {\varLambda}_m(w)\cdot {\omega}_m$	kg	
19	Total body TAG mass (TAG_tot)	${\alpha}_t(w)$	${\alpha}_t(w)$ $=M(w)\cdot {P}_b(w)\cdot {\varLambda}_b(w)\cdot$ 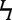 $_b(w)+M(w)\cdot {P}_v\cdot {\varLambda}_v(w)\cdot$ 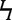 $_v+M(w)\cdot {P}_m(w)\cdot {\varLambda}_m(w)\cdot$ 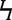 $_m$	kg	
20	TAG availability (TAG_avail)	${\lambda}_t(w)$	${\lambda}_t(w)\sim Uniform(\mathit{\min}=0.5,\mathit{\max}=0.67)$	Prop.	Farmer *et al.* (2018)
21	WE availability (WE_avail)	${\lambda}_w(w)$	*See scenarios* *Table 3*	Prop.	Place (1992), Farmer *et al*. (2018)
22	Starting reserve mass (reserve)	$\tau ({\lambda}_w,w,d=1)$	$\tau ({\lambda}_w,w,d=1\ )={\alpha}_w(w)\cdot {\lambda}_w(w)+{\alpha}_t(w)\cdot {\lambda}_t(w)$	kg	
23	Body condition (rho_w)	${\rho}_w({\lambda}_w,w,d)$	${\rho}_w({\lambda}_w,w,d)=\frac{\tau ({\lambda}_w,w,d)}{M(w)}$	Prop.	
24	Target body condition (rho)	${\rho}_t({\lambda}_w)$	*See WE availability* *Table 1*	Prop.	
25	Lactation body condition threshold (rho_s)	${\rho}_l({\lambda}_w)$	*See WE availability* *Table 1*	Prop.	
26	Central body condition (rho_r)	${\rho}_r({\lambda}_w)$	*See WE availability* *Table 1*	Prop.	
	Foraging time budget				
27	Undisturbed foraging, unmodified (FORcon_u)	${F}_u$	${F}_u=72$	%	Watwood *et al.* (2006)
28	State-dependent foraging (rel_FOR)	$\mu ({\lambda}_w,w,d)$	$\mu ({\lambda}_ww,d)=\frac{1}{1+{e}^{-{\eta}_f\cdot \big(\frac{1.1\cdot {\rho}_{t({\lambda}_w)}}{\rho_{w({\lambda}_w,w,d)}}-1\big)}}$ *Where* ${\eta}_f$ * is a steepness coefficient, set at 21.*	Prop.	De Roos *et al.* (2009), Hin *et al.* (2019)
29	Undisturbed foraging (FORcon)	${F}_b({\lambda}_w,w,d)$	${F}_b({\lambda}_w,w,d)={F}_u\cdot \mu ({\lambda}_w,w,d)$	Prop.	
30	Undisturbed non-foraging active state (NFA_CON)	${R}_b$	${R}_b=2.3$	%	Isojunno *et al.* (2020b)
	Provisioning				
31	Calf age at which milk demand starts to decline (T_n)	${\zeta}_n$	${\zeta}_n=365$	days	Lockyer (1976)
32	Calf age at which milk demand reaches zero (T_l)	${\zeta}_l$	${\zeta}_l=730$	days	Lockyer (1976)
33	Unmodified provisioning (prov_u)	${\sigma}_u$	${\sigma}_u=223.15$	kJ min^−1^	Lockyer (1976)
34	State-dependent Provisioning (prov)	$\sigma ({\lambda}_w,w,d)$	$$\left\{\left.\begin{array}{@{}c@{}}\sigma ({\lambda}_w,w,d)\\\!=\!{\sigma}_u\!\cdot \frac{(1-{\xi}_m)\cdot ({\rho}_w({\lambda}_w,w,d)-{\rho}_l({\lambda}_w))}{({\rho}_r({\lambda}_w)-{\rho}_l({\lambda}_w))-{\xi}_m \cdot ({\rho}_w({\lambda}_w,w,d)-{\rho}_l({\lambda}_w))}\\ {}\cdot \mathit{\min}(1,(\frac{1-\frac{d-{\zeta}_n}{\ {\zeta}_l-{\zeta}_n}}{1-{\xi}_c\cdot \frac{d-{\zeta}_n}{\ {\zeta}_l-{\zeta}_n}\ })\end{array}\right|\sigma \!\ge 0\right\}$$ *Where* ${\xi}_m$ *and* ${\xi}_c$ *are coefficients describing the non-linearity of the state-dependent provisioning and milk demand functions, set at − 2 and 0.9 respectively.*	kJ min^−1^	Hin *et al.* (2019)
	Response to disturbance				
35	Null response intensity	$\varDelta {R}_n$	$\varDelta {R}_n(n=1:1000)=\varDelta {R}_e(1:1000)+\sim Gaussian( mean=0, SD=4)$ *Where ΔR_e_ is a vector of bootstrap samples from the raw empirical response intensities.*	Factor	Isojunno *et al.* (2020a,b)
36	Maximum response intensity	${\varDelta R}_{Max}$	${\varDelta R}_{Max}=17.5$	Factor	Isojunno *et al.* (2020a,b)
37	Average unmodified response intensity (delta_NFA_u)	$\varDelta R$	$\varDelta R=\overline{\varDelta {R}_n}$	Factor	Isojunno *et al.* (2020a,b)
38	Individual difference in response intensity from average (int_var)	$i(w)$	*Needs and ability-based:* $i(w=1:2000)=0$ *Null:* $i(w=2001:3000)=\varDelta {R}_n-\varDelta R$	Factor	Isojunno *et al.* (2020a,b)
39	Response intensity (TME_int)	${\psi}_i({\lambda}_w,w,d)$	*Needs-based:* $\{{\psi}_i({\lambda}_w,w=1:1000,d)\ |\ {\rho}_w({\lambda}_w,w,d)\ge {\rho}_r({\lambda}_w\!)\!\}\!=\!\frac{{\varDelta R}_{Max}-\varDelta R}{\rho_t({\lambda}_w)-{\rho}_r({\lambda}_w)}\!\!\cdot\! ({\rho}_w({\lambda}_w,\!w,\!d)\!-\!{\rho}_r({\lambda}_w)\!)$ *+* $\!\varDelta R$ $\{{\psi}_i\ ({\lambda}_w,w=1:1000,d)|\ {\rho}_w({\lambda}_w,w,d)<{\rho}_r({\lambda}_w)\}=\frac{\ \varDelta R}{\rho_r({\lambda}_w)}\cdot ({\rho}_w({\lambda}_w,w,d)-{\rho}_r({\lambda}_w))$ *+* $\varDelta R$ *Ability-based:* $\{{\psi}_i({\lambda}_w,w=1001:2000,d)\ |\ {\rho}_w({\lambda}_w,w,d)\ge {\rho}_r({\lambda}_w)\}=\frac{-\varDelta R}{\rho_t({\lambda}_w)-{\rho}_r({\lambda}_w)}\cdot ({\rho}_w({\lambda}_w,w,d)-{\rho}_r({\lambda}_w))+\varDelta R$ $\{{\psi}_i\ ({\lambda}_w,w=1001:2000,d)|\ {\rho}_w({\lambda}_w,w,d)<{\rho}_r({\lambda}_w)\}=\frac{\varDelta R-{\varDelta R}_{Max}}{\rho_r({\lambda}_w)}\cdot ({\rho}_w({\lambda}_w,w,d)-{\rho}_r({\lambda}_w))+\varDelta R$ *Null:* ${\psi}_i({\lambda}_w,w=2001:3000,d)=\varDelta R$	Factor	Isojunno *et al.* (2020a,b)
40	Change in foraging due to response (deltaFOR_r)	$\varDelta {F}_r({\lambda}_w,w,d)$	$\{\varDelta {F}_r=\frac{(F_b(\lambda_w,w,d)-({R}_b\cdot ({\psi}_i({\lambda}_w,w,d)+i(w)))-F_b(\lambda_w,w,d)}{F_b({\lambda}_w,w,d)}\ |-1\le \varDelta {F}_r\le 0\}$	Prop.	
41	Response duration, relative to disturbance (resp_dur_u)	${\zeta}_u$	${\zeta}_u=1$	Factor	
42	Response duration (resp_dur)	$\zeta (d)$	$\{\zeta =T(d)\cdot {\zeta}_u|\ 0\le \zeta \le 1140\}$	Minutes day^−1^	
	Energy budget				
43	Patch quality (patch_qual)	$\delta (w,d)$	$\delta (w,d)\sim Gaussian\ ( mean=1, sd=0.42, truncated\ at\ 0,2)$	Factor	Watwood *et al.* (2006)
44	Lipid energy density (lip_eng)	$\theta$	$\theta =42.5\cdot {10}^3$	kJ kg^−1^	Koopman (2018)
45	Energy to structural growth (ED)	$\iota$	$\iota =0$	kJ min^−1^	Lockyer (1976)
46	Field metabolic rate (FMRcon)	$\kappa (w)$	$\kappa =1.0169\cdot {M}^{0.75}$	kJ min^−1^	Noren (2011), Farmer *et al.* (2018)
47	Unmodified energy acquisition (EAeq)	${\chi}_u(w)$	${\chi}_u(w)=\kappa (w)+{\sigma}_u+\iota$	kJ min^−1^	
48	Energy acquisition: baseline (EAcon)	${\chi}_b({\lambda}_w,w,d)$	${\chi}_b({\lambda}_w,w,d)={\chi}_u(w)\cdot \mu ({\lambda}_w,w,d)\cdot \delta (w,d)$	kJ min^−1^	
49	Energy acquisition: response (EAimp)	${\chi}_r({\lambda}_w,w,d)$	${\chi}_r({\lambda}_w,w,d)={\chi}_b({\lambda}_w,w,d)\cdot (1+\varDelta {F}_r({\lambda}_w,w,d))$	kJ min^−1^	
50	Change in energy acquisition during response (deltaEA_r)	$\varDelta {\chi}_r({\lambda}_w,w,d)$	$\varDelta {\chi}_r({\lambda}_w,w,d)={\chi}_r({\lambda}_w,w,d)-{\chi}_b({\lambda}_w,w,d)$	kJ min^−1^	
51	Daily energetic cost of disturbance (DEC)	$\upsilon ({\lambda}_w,w,d)$	$\upsilon ({\lambda}_w,w,d)=\varDelta {\chi}_r({\lambda}_w,w,d)\cdot \zeta (d)$	kJ day^−1^	
52	Daily energy balance (eng_bal)	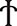 $({\lambda}_w,w,d)$	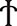 $=\upsilon ({\lambda}_w,w,d)-((\sigma ({\lambda}_w,w,d)-{\sigma}_u)\cdot 1440)+({\chi}_b({\lambda}_w,w,d)-{\chi}_u(w))\cdot 1440$	kJ day^−1^	
53	Energy Reserve (eng)	$\pi ({\lambda}_w,w,d)$	$\pi ({\lambda}_w,w,d)=\tau ({\lambda}_w,w,d)\cdot \theta$	kJ	
54	Reserve mass for following day (reserve)	$\tau ({\lambda}_w,w,d+1)$	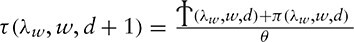	kg	
	State transitions				
55	Maternal death (dead)		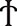 $({\lambda}_w,w,d)+\pi ({\lambda}_w,w,d)\le 0$	Bool.	
56	Provisioning to calf (calf)		$\sigma ({\lambda}_w,w,d)> 0$	Bool.	

Set notation is used to indicate constraints on otherwise continuous relationships and brackets indicate which other values the parameter or variable varies with. d and w values shown are for the full simulations. Initial simulations are for *w* = 1:10^6^ individuals, which only include the calculations up to and including row 23 and only for *d* = 1.

**Table 3:** Simulated scenarios, resulting from combinations of alternative WE availability ranges (λ_w_) and duration of the disturbance source per day (*T*(*d*))

Minutes of disturbance per day; ${T}(d)$	High WE availability; ${\lambda}_w\sim Uniform(\mathrm{0.30,0.50})$	Intermediate WE availability; ${\lambda}_w\sim Uniform(\mathrm{0.05,0.067})$	WE unavailable; ${\lambda}_w=0$
0	WE-40_baseline	WE-06_baseline	WE-00_baseline
480	WE-40_disturbed	WE-06_disturbed	WE-00_disturbed

### Starting values for lipid stores

Starting lipid-store values for initial and full simulations were set following Farmer *et al.* (2018; Table 2, rows 6-22). First, masses for three tissue compartments (blubber, muscle and viscera) were generated by multiplying empirical estimates of the relevant tissue compartment’s proportional mass by the total mass of each individual whale (*M*). The resulting tissue compartment masses were then multiplied by the respective TAG and WE percentages reported in the academic literature to give total lipid store for each compartment. These lipid stores were then partitioned into available reserves versus unavailable structural mass depending on WE availability scenario (see Table 1). Starting values for energy stores ranged from 1.3 to 12.8 × 10^7^ kJ in the full simulations. While we note that these values are different from the simulated values reported by Farmer *et al*. (2018, Fig. 4 therein), our total lipid masses (i.e. before accounting for availability) are consistent with independent empirical measurements of total body lipid in caught sperm whales reported by Irvine *et al*. (2017; Supplementary material S2: Empirical validation of total body lipid stores).

### Lipid-store body condition

Individual body condition (*ρ_w_*) was defined as the lipid reserve mass divided by total body mass (Table 2, row 23). Previous studies have defined reserve mass as the total estimated blubber mass (Christiansen and Lusseau, 2015; Hin *et al.*, 2019; Pirotta *et al.*, 2020). However, as discussed above, composition of sperm whale blubber is variable and there is significant uncertainty surrounding lipid availability. To capture this variation, reserve mass was therefore set to total available lipid mass (see Table 2, row 22). The absolute values presented here may therefore not be directly comparable with those presented elsewhere.

We parameterised the functions governing state-dependent behaviour relative to critical, biologically interpretable thresholds of female body condition. At these thresholds, we expected the balance between risks and rewards to change, thus affecting an individual’s behavioural decision. The first threshold was target body condition (*ρ_t_*), which represents the optimal trade-off between the benefits of increased stored energy and the costs of accumulating additional stores. Such costs may include increased cost of transport (Adachi *et al.*, 2014), increased predation risk associated with intensive foraging (Lima and Dill, 1990) or opportunity costs associated with displaced behavioural budget—for example, time spent foraging instead of socializing or caring for calves. Since there is little benefit for an animal to exceed this threshold, it was set to the 99th percentile of body condition from initial simulations. Foraging effort was set to decrease steeply as body condition increased above this value (see Fig 2A and ‘Resource acquisition’ below).

The second critical body condition threshold was lactation body condition (*ρ_l_*)*.* Above this threshold, fitness is maximised by provisioning milk to the calf. Below this threshold, the individual is expected to prioritize survival over the current reproductive investment (McEwen and Wingfield, 2003). This threshold was therefore used to parameterize the rate of energy delivery to the calf (see ‘Provisioning of offspring’ and Fig. 2B). To our knowledge, no systematic data are currently available on the body composition of starved or emaciated sperm whales. A population of an income breeding species is likely to include a small number of females which, having brought a calf to term, have inadequate surplus energy to support it (Stephens *et al.*, 2014). Thus, lactation body condition was set at the 5th percentile of body condition from initial simulations.

Finally, the third critical threshold was central body condition (*ρ_r_*). This represents the average body condition of the population and was used to parameterize state-dependent disturbance responses (see ‘Behavioural response to disturbance’ and Fig. 2C). It was set to the mean body condition from initial simulations.

### Provisioning of offspring

Long-lived, iteroparous species that prioritize survival over reproduction, such as sperm whales, are expected to balance their investment in each reproductive event with their own risk of starvation (Whitehead, 2003). Milk provisioning to a dependent calf was therefore modelled as a function of body condition and calf age (De Roos *et al.*, 2009; Hin *et al.*, 2019). Unmodified provisioning (*σ_u_*) was set from Lockyer (1976; Table 2, row 33) and was then modified depending upon female body condition and calf demand (Table 2, row 34; Fig. 2B). Female provisioning (*σ*) was equal to unmodified provisioning at central body condition (*ρ_r_*). As individual body condition reduced, provisioning was progressively reduced and, at values less than or equal to lactation body condition (*ρ*_*l*,_), ceased entirely. Calf demand was set to decline with age, such that it was equal to 1 for the first year and then declined at an increasing rate until it ceased entirely after 2 years. As Hin *et al.* note, empirical estimates of these parameters do not exist for any odontocete species and would be extremely difficult to collect. We have therefore followed their approach of developing functional forms that qualitatively reflect our theoretical expectations (Hin *et al.*, 2019, particularly supp. info. therein).

For simplicity, we did not model the effects of reduced provisioning on calf survival. Doing so accurately would require consideration not only of the body condition and energy storage capabilities of the calf but also those of potential allomothers in the social group (Gero *et al.*, 2009), which is beyond the scope of this study. Rather, we report changes in energy provisioned and the number of days with no provisioning, which provides a starting point for future efforts to model calf survival.

### Resource acquisition

Under undisturbed conditions, sperm whales spend most of their time in behaviours associated with foraging: vertical transit, layer-restricted search and surface intervals; spent recovering and replenishing oxygen reserves (Watwood *et al.*, 2006; Isojunno *et al.*, 2020b). The undisturbed proportion of time spent foraging (*F_u_*; Table 2, row 27) was therefore set from the average percentage of time spent in foraging dive cycles reported for the Atlantic population by Watwood *et al*. (2006).

Females were assumed to acquire energy at a rate sufficient to meet metabolic demands when undisturbed. Lockyer (1976) estimated that, in order to remain at equilibrium, lactating females must increase their energy acquisition rate by 32%–63% depending on age class. As the time allocated to foraging is normally very high in sperm whales (72%–97%; Watwood *et al.*, 2006; Isojunno *et al.*, 2020b), we assumed that lactating females forage close to their maximum capacity and there is negligible scope to increase foraging to compensate for worsening condition. Unmodified energy acquisition (χ_u_) was therefore assumed to be equal to the sum of unmodified provisioning (*σ_u_*) and field metabolic rate (κ; Table 2, row 47).

A state-dependent foraging effort parameter (*μ*) was added to prevent body condition from increasing above realistic bounds after provisioning ceased. Following Hin *et al*. (2019), this was modelled as a decreasing sigmoid function (Table 2, row 28; Fig. 2A), such that it decreased as individual body condition (*ρ_w_*) approached target body condition (*ρ_t_*), essentially offsetting the reduction in energy invested in provisioning towards the end of lactation. As with the state-dependent provisioning function, a lack of empirical data required a qualitative parameterization of the function. Daily stochastic variability in patch quality (*δ*) was modelled by drawing a value for each whale from a normal distribution centred on 1 and with standard deviation equal to the coefficient of variation in number of buzzes per dive from Watwood *et al.* (2006; Table 2, row 43). Baseline energy acquisition (*χ_b_*) was then calculated by multiplying unmodified energy acquisition by foraging effort and patch quality (Table 2, row 48).

When the sum of the energy available from both foraging and lipid stores was less than that required for metabolism on a given day, the female was assumed to have died of starvation and was removed from the simulation. For simplicity and ease of interpretation, other sources of mortality (predation, anthropogenic, etc.) were not included.

### Behavioural response to disturbance

Behavioural responses to disturbance were specified based on the results of controlled experiments exposing tagged whales to naval sonar (Isojunno *et al.*, 2020a,b)*.* Response intensity was defined as the increase in ‘non-foraging active state’ (NFA), during which echolocation ceases but the animal remains active rather than recovering at the surface or exhibiting stereotypical resting behaviour (Isojunno and Miller, 2015). In controlled-exposure experiments, switching to the non-foraging active state in response to sonar has only been observed from foraging-related behavioural states (Isojunno *et al.*, 2020b). Increases in NFA were therefore assumed to cause an equal decrease in foraging. Response intensity was parameterised using the observed per-session response intensities during exposures exceeding 137 dB re 1 μPa^2^ s^−1^ (Isojunno *et al.*, 2020a; our reanalysis, hereafter ‘Empirical response intensities’). This sound exposure threshold was chosen because it is the lowest level at which responses potentially impacting vital rates were observed in a qualitative analysis of the same data (Curé *et al.*, 2021).

Three alternative response strategies were considered (Fig. 2C). Under the ‘ability-based’ strategy, individuals increased their risk-taking (and therefore responded less) as their body condition increased. Conversely, under the ‘needs-based’ strategy, individuals responded more as their body condition increased. Finally, under the ‘null’ strategy, response costs were independent of body condition.

Empirical studies show high variability in response intensity (Harris *et al.*, 2018). This is likely to be driven by a combination of stage- and state-dependent strategies, consistent individual differences and various environmental factors such as predator presence and patch quality. The objective of this study was to explore the potential effects of state-dependent mechanisms. To obtain a maximum plausible effect size for state-dependent strategies, we therefore assumed that for the needs- and ability-based strategies the entire range of empirical responses resulted from deterministic body condition-dependent behavioural strategies. In the null scenario, the same range of responses was instead assumed to arise exclusively from stochastic differences in sensitivity, which remained constant within individuals.

To make meaningful comparisons across the three strategies, range and mean response intensity were constrained across all three scenarios as follows. For the null scenario, a proposed distribution of response intensity was generated by sampling from the empirical response intensities with replacement, then adding normally distributed pseudorandom noise to the samples to generate intermediate values (Table 2, row 35). This distribution was then truncated at the minimum and maximum values from the empirical dataset.

For the needs- and ability-based strategies, response functions were parametrised such that over the range of possible body conditions simulated (0 − ~*ρ_t_*), conditional on a given WE scenario, the function produced the full range of response intensities observed empirically by Isojunno *et al.* (2020a,b), since individuals are expected to optimize state-dependent strategies by reference to their risk of starvation and that where body condition (*ρ_w_*) is equal to central body condition (*ρ_r_*), response intensity (${\psi}_i$) is equal to the mean response intensity under the null scenario, to ensure the generalization of results between strategies. The asymmetrical state-dependent response intensity functions shown in row 39 of Table 2 and Fig. 2C were therefore chosen as the simplest solution to meet both conditions.

### Reduction in energy acquisition due to responses

Response costs ($\upsilon$) were calculated at a daily scale, based on the methods described by Christiansen and Lusseau (2015). The effects of disturbance on foraging were calculated by taking the baseline rate of energy acquisition (${\chi}_b$), reducing it proportionately with the change in time allocated to foraging ($\varDelta F_r$) and taking the difference to give the change in rate of energy acquisition per minute of response (*Δχ_r_*; Table 2, row 50). This was then multiplied by the response duration (*$\zeta $*), which here was assumed to be equal to disturbance source duration, to give daily response cost ($\upsilon$; Table 2, row 51). Energetic costs of increased locomotion during response are thought to be low compared with those of lost feeding opportunities, unless the change in locomotion is dramatic (Czapanskiy *et al.*, 2021). Isojunno *et al.* (2020b) found no strong support for a change in activity level during response, so field metabolic rate (*κ*; Table 2, row 46) was assumed to be unchanged during response.

### Simulation procedures

#### Scenarios of WE availability

**Figure 3:**
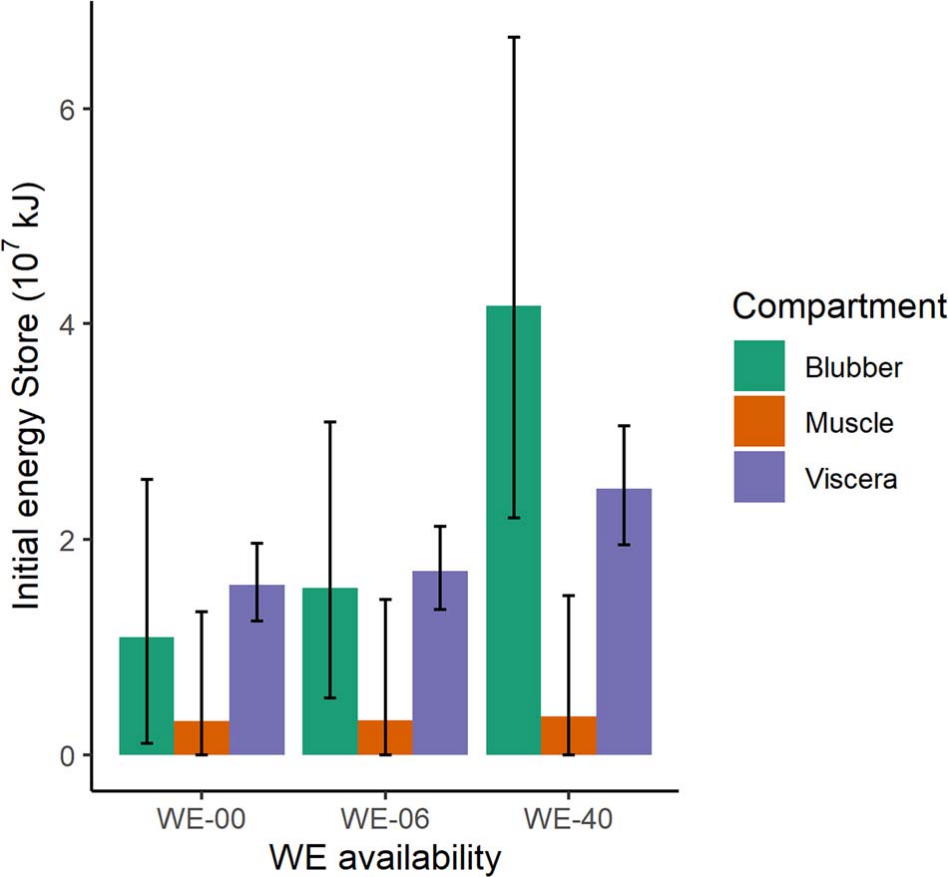
Starting energy stores for different compartments under alternative simulation scenarios. Error bars show 95% range.

As discussed above, there is uncertainty about the catabolic availability of WE lipid stores, that is, to what extent they truly represent energy reserves versus structural mass. To explore the potential consequences of this uncertainty, WEs were partitioned to reserve and structural mass differently across three scenarios, corresponding to alternative physiological hypotheses (Fig. 3). The WE-00 scenario assumed no WE availability, reflecting the hypothesis that these lipids serve only a specialised diving function rather than acting as an energy store. The WE-40 scenario assumed that 30%–50% of WEs were available, which corresponds to the highest plausible values modelled by Farmer *et al.* (2018) and represents a mobilization efficiency comparable with that of TAGs. The WE-06 scenario assumed a distribution of availability between 5% and 6.7%, reflecting the hypothesis that WEs serve an intermediate physiological role and can only be mobilised incompletely or with an efficiency comparable with that of other mammals (Place, 1992). The acoustic fats in the spermaceti organ and junk are thought to be metabolically unavailable (Koopman, 2018) and were thus treated as structural mass across all scenarios.

#### Simulation of exposure to disturbance

Full simulations were carried out under both baseline and disturbed conditions. The baseline scenario included no disturbance. For the disturbed scenario, an extreme disturbance source duration (*T = 460* [8 hours] per day for all days) was simulated to demonstrate the emergent properties of the system.

#### Implementation

The model equations described above and in Table 2 were implemented in the R programming language and software environment for statistical computing (version 4.0.5; R Core Team, 2021) and iteratively run over the simulation period for various disturbance and WE availability scenarios shown in Table 3. The fully commented model simulation code is freely available through the open science framework (Burslem *et al.*, 2022).

#### Sensitivity analysis

We conducted a sensitivity analysis to check that our results were not overly influenced by non-empirical assumptions, relative to the daily disturbance duration (*T*). Sensitivity was assessed by changing each parameter by 10% while holding all others constant at the values reported in Table 2. Detailed sensitivity analysis methods and results are reported in the supplementary information (S1: Sensitivity analysis).

## Results

### Baseline behaviour of the model

**Figure 4:**
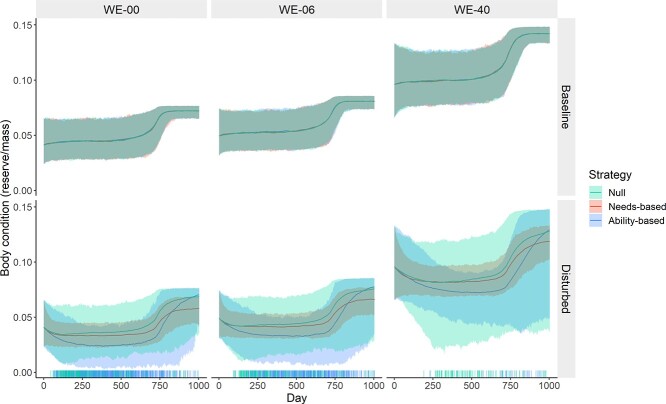
Body condition over the course of the 1004-day simulations, by disturbance (rows; applied on all days) and WE availability scenario (columns). Calf demand was specified to decline with age from Day 365 and cease completely at Day 730. Solid lines show mean daily values, and shaded areas show 95% range. Rug plots show maternal deaths. Colours indicate response strategy. Dead whales are not included in calculations of daily means and ranges.

**Figure 5:**
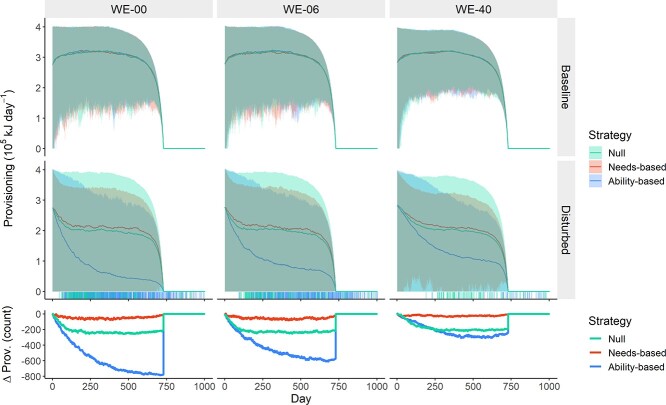
Variation in provisioning over the course of the 1004-day simulations, by disturbance (rows; applied on all days) and WE availability scenario (columns). Calf demand was specified to decline with age from Day 365 and cease completely at Day 730. Rug plots show maternal deaths, lines show the mean daily values and shaded areas show 95% range. The bottom panel shows the change in the number of whales provisioning between baseline and disturbed scenarios. Colours indicate response strategy. Dead whales are treated as zeroes.

Under baseline scenarios with no disturbance (Figs 4 and 5, Baseline) body condition and energetic balance were determined by life history processes built into the model. During lactation, individual body condition tended towards central body condition (*ρ_r_*), driven by state-dependent provisioning. As provisioning progressively reduced following Day 365 (Figs 2B and 5, Baseline), body condition increased as progressively more acquired energy was allocated to maternal reserves (Fig. 4, Baseline). Finally, energy acquisition began to reduce due to decreasing state-dependent foraging effort, reaching a new equilibrium close to target body condition (*ρ_t_*; Fig. 4, Baseline).

### Body condition and behavioural responses

**Figure 6:**
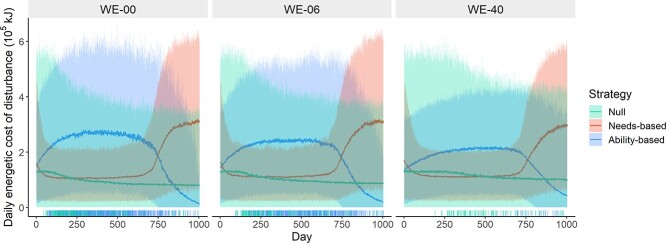
Daily energetic costs of disturbance response. Solid lines show the mean daily values for living whales, and shaded areas show 95% range. Disturbance was applied on all days. Calf demand was specified to decline with age from Day 365 and cease completely at Day 730. Rug plots show maternal deaths. Colours indicate response strategy. Whales that died are not included in calculations of the mean values for later days.

Under all WE scenarios and behavioural strategies, individuals bore costs of behavioural response to disturbance (mean daily response costs, kJ × 10^−4^: WE-00 = 16.7, WE-06 = 16.2 WE-40 = 15.0; Fig. 6) and body condition was consequently reduced relative to baseline (mean reductions in body condition *ρ_w_* for living animals: WE-00 = 38.9%, WE-06 = 30.9%, WE-40 = 20.6%; Fig. 4). However, both the size and variability of response costs varied strongly depending on the strategy followed. Under the null strategy, average response costs stayed relatively constant both between WE scenarios and within simulation runs, with daily average decreasing slightly over the course of the simulation as the most sensitive individuals died and were removed (Fig. 6). For animals following the needs-based strategy, daily response costs ($ v $) were comparatively low during nursing but increased dramatically as body condition improved following weaning, leading to an overall average 23.6%–38.3% higher than for the null strategy. Animals following the ability-based strategy incurred high costs during nursing. For those that survived, costs reduced dramatically after weaning but were still 42.4%–104.4% higher overall, relative to the null strategy.

Despite high overall response costs, the needs-based strategy resulted in relatively small reductions in body condition (Table 4), because reduction in body condition led to a reduction in response intensity, rapidly leading to a stable equilibrium between the two; the level at which this equilibrium was reached was determined by the level of disturbance and other energetic demands on the individual (e.g. lactation). In contrast, under the ability-based strategy, as body condition declined the cost of disturbance increased, which in turn decreased body condition further. This led to positive feedback between body condition decline and response intensity. Individuals that started the simulation with greater energy stores benefited from reduced response costs and thus tended to stay in good condition, provided stochastic variation in patch quality did not cause their body condition to drop below central body condition (*ρ_r_*) and were able to fully recover after weaning (Fig. 4). These mutually reinforcing differences in body condition and response intensity led to the high magnitude and variability in body condition reduction seen in this group (Fig. 4; Table 4). The effect of this feedback cycle was stronger under lower WE availability scenarios, because total individual energy store was smaller relative to the daily energetic budget, resulting in steeper slope of the assumed state-dependent response functions (*ψ*) and more rapid positive feedback within simulations (Table 4; Fig. 4, see also sensitivity analysis S1).

**Table 4:** Energetic, reproductive and survival outcomes from the disturbance scenarios, summarised across the 1000 individuals simulated per strategy

WE availability scenario	Response strategy ($\psi$)	Average energetic cost of response^a^ (kJ 10^4^ day^−1^;$\upsilon$)	Reduction in body condition^b^ (*ρ_w_*; %)	Total reduction in provisioning (kJ 10^7^)	Increase in total days without provisioning^c^	Maternal death rate (%)
WE-00	Ability-based	23.1 ± 2.8	66.0 ± 25.8	15.5 ± 4.3	416.2 ± 186.6	73.1
WE-00	Needs-based	15.7 ± 1.6	24.1 ± 6.5	6.3 ± 2.8	34.0 ± 37.0	0
WE-00	Null	11.3 ± 6.5	25.6 ± 32.1	7.3 ± 7.9	156.0 ± 234.6	16.8
WE-06	Ability-based	21.0 ± 2.6	47.6 ± 26.6	13.8 ± 4.4	318.1 ± 183.1	47.0
WE-06	Needs-based	15.9 ± 1.6	21.2 ± 5.5	6.4 ± 2.7	38.2 ± 37.2	0
WE-06	Null	11.8 ± 6.8	23.1 ± 29.6	7.2 ± 7.8	153.0 ± 230.2	14.2
WE-40	Ability-based	17.5 ± 2.0	24.3 ± 12.0	11.1 ± 3.8	155.5 ± 133.7	3.7
WE-40	Needs-based	15.2 ± 1.7	17.8 ± 5.2	6.1 ± 2.7	16.0 ± 27.0	0
WE-40	Null	12.3 ± 5.9	18.9 ± 24.6	6.8 ± 7.5	127.1 ± 218.5	8.7

^a^Response costs averaged for each whale over all the days that the individual is alive, reported as group mean and SD.

^b^Reduction in body condition relative to baseline, expressed as mean and SD percentage change in the area under the curve of simulated individual body condition.

^c^Increase in the number of days without milk provisioning relative to baseline, reported as group mean and SD.

### Effects of disturbance on provisioning

The mother’s prioritization of her own survival meant that, once her body condition began to decline, much of the energetic cost of disturbance was passed on to the calves in the form of reduced provisioning. For this reason, provisioning was reduced by disturbance across scenarios (mean reductions in total provisioning: WE-00 = 45.4%, WE-06 = 42.6%, WE-40 = 37.6%). The amount by which provisioning was reduced varied between strategies, with reduction in total provisioning improving under the needs-based strategy across WE scenarios (by 10.0%–13.0%, depending on WE availability) and worsening under the ability-based strategy (by 63.1%–113.0%) relative to the null strategy (see Fig. 5; Table 4).

The number of days on which any milk was provided to the calf followed similar patterns (Fig. 5, bottom row). On average the increase in the number of days without provisioning under disturbance was 75.0%–87.4% lower for the needs-based strategy relative to the null strategy. For the ability-based strategy, the increase was 22.4%–166.8% greater depending on WE availability. Needs-based provisioning also resulted in shorter provisioning interruptions. Only 2.8%–5.0% of animals following the needs-based strategy had at least one run of continuous provisioning cessation that exceeded 40 days in length during the first 364 days, when the calf is assumed to be completely dependent on maternal provisioning. After removing adult females that died during the simulation, the corresponding percentages for the null and ability-based strategies were 12.9%–16.2% and 26.6%–46.1%, respectively.

### Mortality of adult females

Deaths only occurred in scenarios with disturbance. No animals following the needs-based strategy died, as response costs decreased as body condition declined and plateaued at a level at which all individuals were able to offset the energetic costs of disturbance through reduced provisioning. For animals following the null strategy there was no stabilizing mechanism and a small and relatively consistent number of the most responsive animals (8.7%–16.8%) died across all WE availability scenarios. For the ability-based strategy, costs of disturbance increased progressively as body condition declined and, by the time whale body condition *ρ_w_* had reached lactation threshold condition *ρ_l_*, even total cessation of provisioning to the calf was often insufficient to compensate. For 3.7%–73.1% of whales, depending on WE availability (Table 4), reduction in foraging due to disturbance led to rapid depletion of reserves and ultimately death.

In scenarios assuming lower WE availability, deaths occurred both at a higher rate (Table 4) and earlier in time (mean day at death: WE-00 = 395.5, WE-06 = 487.7, WE-40 = 563.6). This was partly due to lower absolute energy store and therefore shorter time to starvation. As described above, decreased WE availability also amplified the effects of state-dependent strategies. The mortality rate for animals with an ability-based strategy decreased from 73.1% to 3.7% between the WE-00 and WE-40 scenarios, while the corresponding reduction for the null strategy was only from 16.8% to 8.7%. While the ability-based strategy accounted for most maternal deaths under the WE-06 and WE-00 scenarios, the null strategy resulted in more deaths under the most optimistic WE-40 scenario (Table 4).

## Discussion

This study used simulation modelling to explore the potential effects of body condition-dependent behavioural response modification on the consequences of disturbance. The simulations show strikingly different consequences of disturbance on mortality and calf provisioning, depending on which condition-dependent response strategies the animals followed and on the energetic availability of WE lipids. Compared with scenarios where individual responses were independent of body condition, a needs-based strategy avoided maternal death and resulted in few long periods of provisioning cessation. In contrast, an ability-based strategy exaggerated both consequences. Reduced WE availability resulted in reduced time to starvation and greater sensitivity to responses mediated by body condition.

### Effects of disturbance on vital rates

The extent to which disturbance translated into effects on vital rates varied dramatically between body condition-dependent response strategies. Under the needs-based strategy (i.e., animals in better condition responding more intensely), the highest response costs were borne during times of favourable body condition. While this had the effect of limiting post-weaning recovery in body condition, which may be expected to impact future population fecundity, the most severe consequences were avoided; deaths were completely absent, and periods of provisioning cessation were relatively brief and infrequent. Conversely, under the ability-based strategy (i.e., animals in worse condition responding more intensely), a positive feedback cycle developed between decreasing body condition and response intensity. As a result, when body condition had declined below the lactation threshold (ρ*_l_*), even the total cessation of nursing that followed was often insufficient to compensate for the increased cost of response. For long-lived species with slow life history traits, such as sperm whales, the high number of maternal deaths that resulted would be expected to have a significant impact on population status (Heppell *et al.*, 2000; Oli, 2004; Stahl and Oli, 2006).

There were dramatic effects of disturbance on provisioning, because we specified that mothers would preferentially reduce provisioning rather than allow their own body condition to decline to levels that could threaten their survival. These results demonstrate the potential for state-dependent provisioning to mask the population-level effects of disturbance if only adults are studied. The relationship between body condition and provisioned energy has not been investigated for any odontocete, since it is usually impractical to confirm milk delivery or estimate volume (Gero and Whitehead, 2007). However, aerial photogrammetry has recently been used successfully to quantify changes in body condition during foraging interruption for odontocetes (Currie *et al.*, 2021) and assess how female body condition drives calf development in baleen whales (Christiansen *et al.*, 2016, 2018). Similar data on calf development and maternal condition could be collected for odontocetes, ideally under both natural and disturbed conditions.

Our model deliberately ignored allonursing, which may be an important factor affecting calf survival in social mammals such as sperm whales. Social network analysis supports the hypothesis that alloparental care is a strong driver of associations and has an important role in the evolution and maintenance of sperm whale social structure (Gero *et al.*, 2013). However, it is unknown whether the amount of milk provisioned is sufficient to constitute an important source of energy for the calf or whether allonursing merely serves to maintain social bonds evolved to minimize the risk of predation. Patterns of allonursing vary between populations and may represent an important source of variability in resilience to disturbance (Konrad *et al.*, 2019). For example, the number of older individuals in a group may significantly influence calf survival within that group despite, or even because of, those individuals having no calves of their own. Gero *et al.* (2009) investigated the relationship between allonursing and relatedness and concluded that, at least over short timescales, rates of allonursing may sometimes exceed rates of nursing from the mother, with allonursing usually but not exclusively coming from closely related individuals. Calf survival may therefore depend on the number, relatedness and nutritional status of members of the social group. Systematic data on how rates of allonursing and associated calf development vary with the condition of both mothers and allomothers could therefore greatly benefit our understanding of population-level resilience to disturbance.

### Influence of body condition on behavioural responses

We modelled relatively simple hypothetical relationships between body condition and responsiveness and assumed that needs-based and ability-based strategies were mutually exclusive, though more complex relationships are likely to exist in nature (Luttbeg and Sih, 2010). Understanding these mechanisms may be important for the correct interpretation of empirical results. For example, responsiveness may have a negative ability-based relationship with body condition only when body condition is above a threshold at which the risk of predation outweighs that of starvation (Sih *et al.*, 2015). Below this threshold, responsiveness could be positively related to body condition, reflecting the decrease in net benefit of foraging cessation when body condition is poor. Under such a strategy, responsiveness would be low not only when body condition was above average, but also when it was very low. This would make responses observed experimentally difficult to interpret unless body condition data were also collected, because observed responsiveness may be low precisely because the individual has already been highly impacted by disturbance. This highlights the need for a mechanism-based interpretation of observed response intensity in controlled-exposure experiments (e.g. habituation vs tolerance; Beale and Monaghan, 2004b; Bejder *et al.*, 2009; Wensveen *et al.*, 2019).

Distinguishing between alternative state-dependent mechanisms in nature will require the collection of empirical data on the relationship between body condition and behavioural responses to disturbance in marine mammals. Such data are not straightforward to collect. Behavioural responses may be influenced by a range of contextual factors, both intrinsic and extrinsic (Beale and Monaghan, 2004a; Harris *et al.*, 2018). Intrinsic factors include not only body condition, but also motivational state and repeatable individual differences in boldness and speed of learning. Long-term repeatable differences are difficult to study in many free-ranging marine mammals, particularly large marine-obligate cetaceans such as sperm whales. However, advances in tag data processing methodologies do allow for simultaneous quantification of body condition proxies and behavioural disturbance (Biuw *et al.*, 2003; Miller *et al.*, 2004; Isojunno and Miller, 2015; Aoki *et al.*, 2021), supporting detailed study of their interplay (Beltran *et al.*, 2021; Siegal *et al.*, 2022). Contemporary developments in tag system architecture could allow the onboard processing of raw sensor data into summary body condition metrics (Williams *et al.*, 2021). This would lead to the observation of patterns of behaviour and body condition over broader spatial and longer temporal ranges than is currently possible, potentially allowing them to be distinguished from repeatable individual differences. Meanwhile, continued research into anti-predator behaviour of marine mammals under undisturbed conditions will be valuable for determining the relative plausibility of alternative state-dependent response strategies. If the empirical evidence supports ability-based strategies in cetaceans, as in Siegal *et al.* (2022), it will be important to ascertain by what mechanism improved condition confers anti-predator advantages. For example, neutral buoyancy or enhanced diving capability could aid vertical escape or use of refugia at depth by individuals in good condition (Miller *et al.*, 2012). Improved understanding of these mechanisms may, in turn, help to inform exploration of analogous state-dependent strategies in simulation models, including how these strategies may affect disturbance responses. Ultimately, future PCoD models could be developed to explicitly include these strategies, illuminating potential non-linear effects and improving our ability to model the consequences of disturbance on marine mammal populations and set precautionary thresholds.

### Energy store availability

Average energy available from blubber at the start of the simulation increased by a factor of 3.8 when WEs were 30%–50% available, compared with completely unavailable (Fig. 3). Assuming no food intake, this translated to an increase in mean time to starvation of 134% (31.9 vs 13.6 days). In addition, in our simulations reduced WE availability resulted in greater sensitivity to body condition-dependent response costs. Considerable work has been done towards measuring total blubber lipid content in deep-diving cetaceans (Evans *et al.*, 2003; Kershaw *et al.*, 2019), but total lipid content may mask significant variability in lipid class composition, and therefore total available energy store (Koopman, 2018). Few studies have attempted to quantify variation in blubber composition in sperm whales, and those that are available do not use standardised sampling locations in the body (Lockyer, 1991). New data quantifying how lipid class composition changes with life history stage and nutritional stress are therefore needed to understand the resilience of deep-diving odontocetes to nutritional stress resulting from both natural and anthropogenic causes.

## Conclusions

State-dependent risk-taking is well documented in animal behaviour but not commonly implemented in models of the energetic and population consequences of disturbance. The simulation results presented here demonstrate that alternative state-dependent behavioural responses can lead to substantially different outcomes under conditions of sustained disturbance due to feedback effects. We explored such effects by specifying alternative relationships between individual body condition and response intensity in terms of foraging cessation, but similar state-dependent effects of disturbance could be present in other behavioural contexts, such as social behaviour, or as a function of other physiological state variables, such as stress hormone status. Such response modifications should therefore be incorporated into future models for the population consequences of disturbance, where justified by empirical data on the behavioural ecology of the species of interest.

## Supplementary material

Supplementary material is available at *Conservation Physiology* online.

## Data availability

The data and code necessary to reproduce the results presented in this article are freely available via the open science framework (OSF): https://doi.org/10.17605/OSF.IO/NJ83K.

## Funding

This work was supported by the United States Office of Naval Research (awards N00014-17-1-2757 and N00014-19-1-2479).

## Supplementary Material

supp_data_coac069
