# Supplementary material for: Modelling the impact of condition-dependent responses and lipid-store availability on the consequences of disturbance in a cetacean
Source: Conserv Physiol. 2022 Nov 17;10(1):coac069. doi: 10.1093/conphys/coac069 (PMC9672687; doi:10.1093/conphys/coac069)
Supplement: supp_data_coac069 [file supp_data_coac069.zip › Burslem-etal_S1_sensitivity_analysis_FINAL_SUBMITTED.docx]

**S1: Sensitivity analysis**

We conducted a sensitivity analysis to check that our results were not overly influenced by non-empirical assumptions. We considered the model sensitive to a parameter if changing the parameter by 10% resulted in a >10% change in any of the outcome variables relative to the control run (i.e. without any parameter values changed) under any strategy. We conducted sensitivity runs assuming WE availability in the WE-06 scenario, with two alternative control disturbance source durations (*T)*. The first was 8 h per day, as reported in the paper. However, this is a deliberately extreme value, which could lead to some effects being at or close to their constraints and thus mask sensitivity at intermediate values. We therefore repeated the analysis using a more moderate control disturbance duration of *T* = 6 h per day.

Only the ability-based strategy models resulted in sensitivities above the 10% threshold (Figs S1-S2). Of the assumptions in our model that were not based on empirical evidence, the model was sensitive to the lactation threshold *ρ_l_*. This is unsurprising and reinforces our conclusion that improved data on the relationship between body condition and nursing and/or calf development in sperm whales would improve the predictive value of the model. Increase in *T* resulted in increased response costs and decreased body condition and provisioning. The relative sensitivity of the ability-based strategy is also consistent with our understanding of the emergent properties of the model: namely that ability-based responses exacerbate effects of disturbance through non-linear feedback processes, while needs-based responses reduce them. Sensitivity of daily response costs *υ* to increase in *T* was high for both control values of *T*, while provisioning *σ* and body condition *ρ_w_* were more sensitive where control *T* = 8 h. This suggests that, at 8 h per day, a high proportion of individuals were indeed responding at their maximum, with costs of further increase in disturbance passed on to body condition and provisioning.

Table 1: Notation used in the sensitivity analysis.

| **Notation** | **Description** | **Type** |
| --- | --- | --- |
| *d* | Day index | Input |
| *ξ_c_* | Steepness parameter of calf demand decline | Input |
| *ξ­_m_* | Steepness parameter of maternal provisioning decline | Input |
| *ζ_n_* | Duration of nursing as calf’s only energy source | Input |
| *ζ_l_* | Duration of lactation | Input |
| *δ_sd­­_* | Variability in patch quality | Input |
| *ρ_l_* | Body condition lactation threshold | Input |
| *ρ_t_* | Target body condition | Input |
| *T(d)* | Daily disturbance duration | Input |
| *ρ­_w_* | Average body condition | Output |
| *σ* | Sum of total provisioning | Output |
| *υ* | Average daily costs of disturbance | Output |


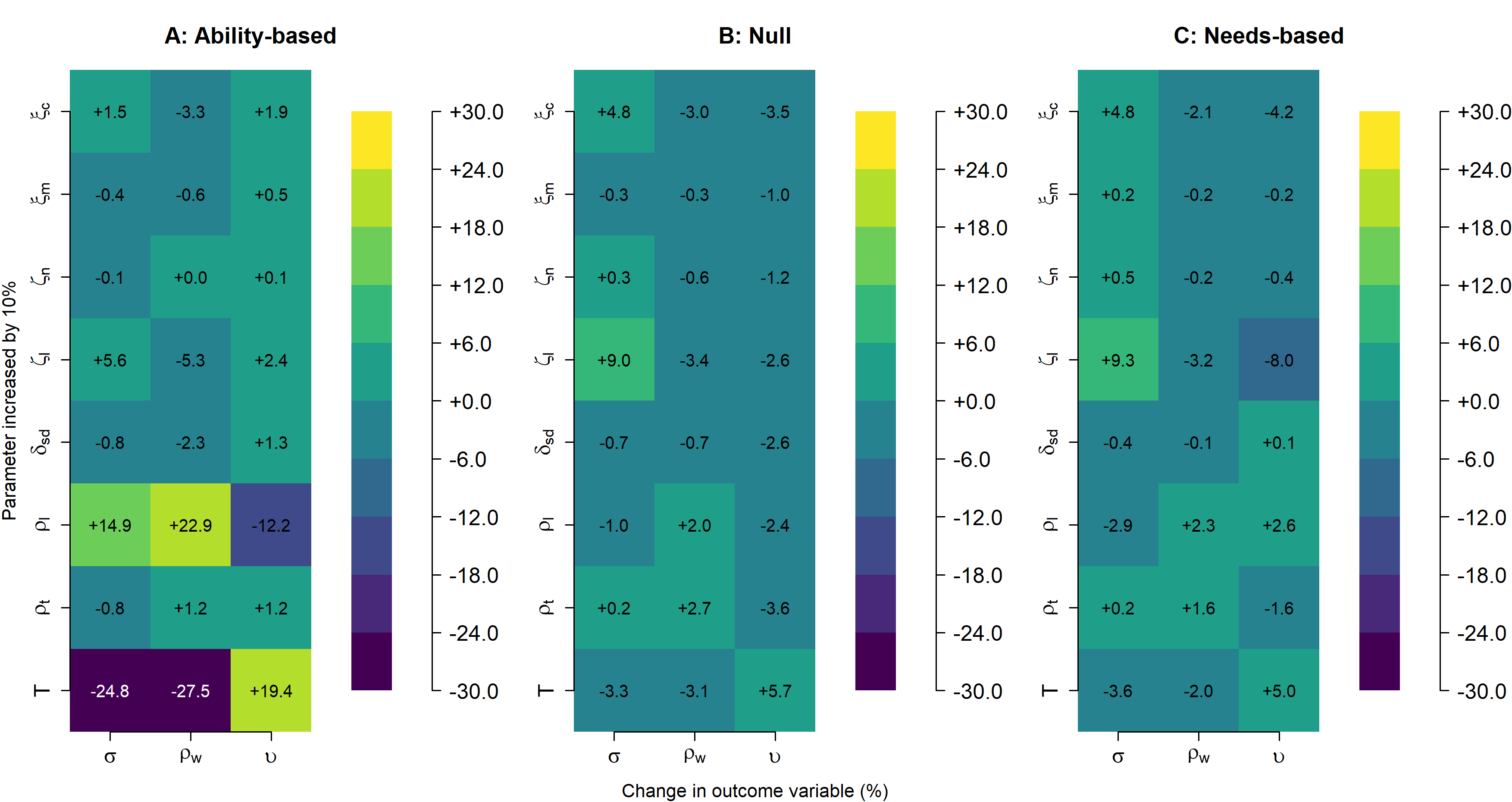


Figure S1: Sensitivity analysis for disturbance duration T= 8h /day. X-axis shows continuous model outcome variables as reported in Table 4 of the paper. Y-axes show input parameters. Values and colours indicate the change in X when Y is increased by 10% and all other variables are held constant at the values reported in the main text.


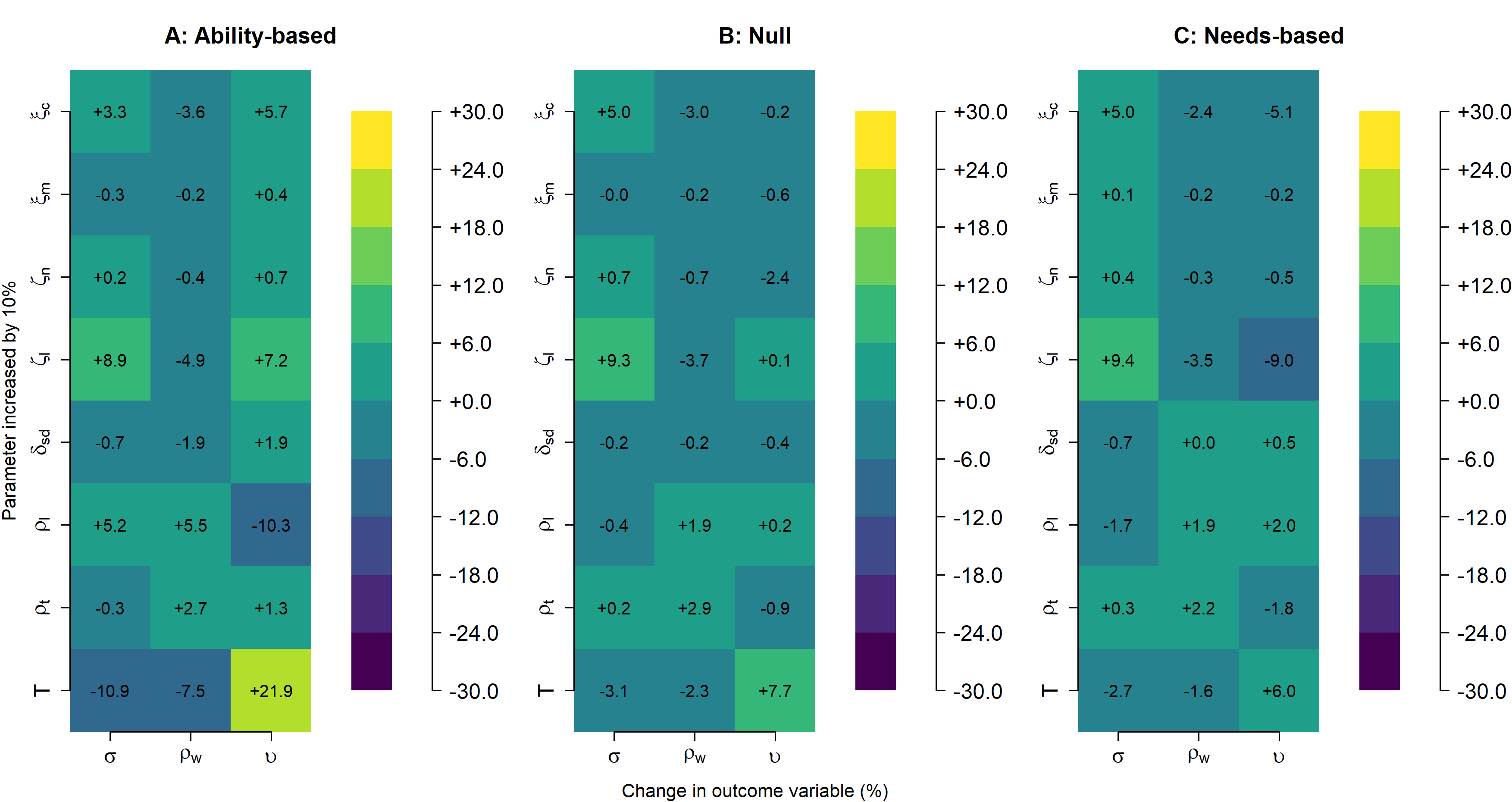


Figure S2: Sensitivity analysis for disturbance duration T= 6h /day. X-axis shows continuous model outcome variables as reported in Table 4 of the paper. Y-axes show input parameters. Values and colours indicate the change in X when Y is increased by 10% and all other variables are held constant at the values reported in the main text.
