# Supplementary material for: Modelling the impact of condition-dependent responses and lipid-store availability on the consequences of disturbance in a cetacean
Source: Conserv Physiol. 2022 Nov 17;10(1):coac069. doi: 10.1093/conphys/coac069 (PMC9672687; doi:10.1093/conphys/coac069)
Supplement: supp_data_coac069 [file supp_data_coac069.zip › Burslem-etal_S2_lipid-validation_FINAL_SUBMITTED .docx]

**S2: Empirical validation of total body lipid stores**

Total body lipid store is a critical component of our model but is estimated from parameters based on a small sample of measurements (e.g. visceral lipid content from one whale; Lockyer, 1991). We therefore validated total lipid store as a function of body length by comparing the values for our simulated whales against the model fitted to catch yield data by Irvine *et al*. (2017). Total body lipid was calculated for simulated animals by summing the lipid stores from all estimated lipid store compartments (viscera, blubber and muscle) using the methods described in the main paper, but without applying the availability parameters. This was then converted to volume using the specific gravity for ‘whale oil’ (Dieterichs, 1906) and plotted against Irvine *et al.*’s empirical model for comparison. The catch data include oil from the spermaceti organ, while the simulated lipid stores do not. We therefore plotted Irvine *et al.*’s model both with and without a 11% reduction in total lipids to account for the contribution spermaceti is thought to make to total whale oil yield (Clarke, 1978). The resulting plots are shown below (Fig. S3) and demonstrate that the total body lipid stores simulated here are consistent with those observed empirically.


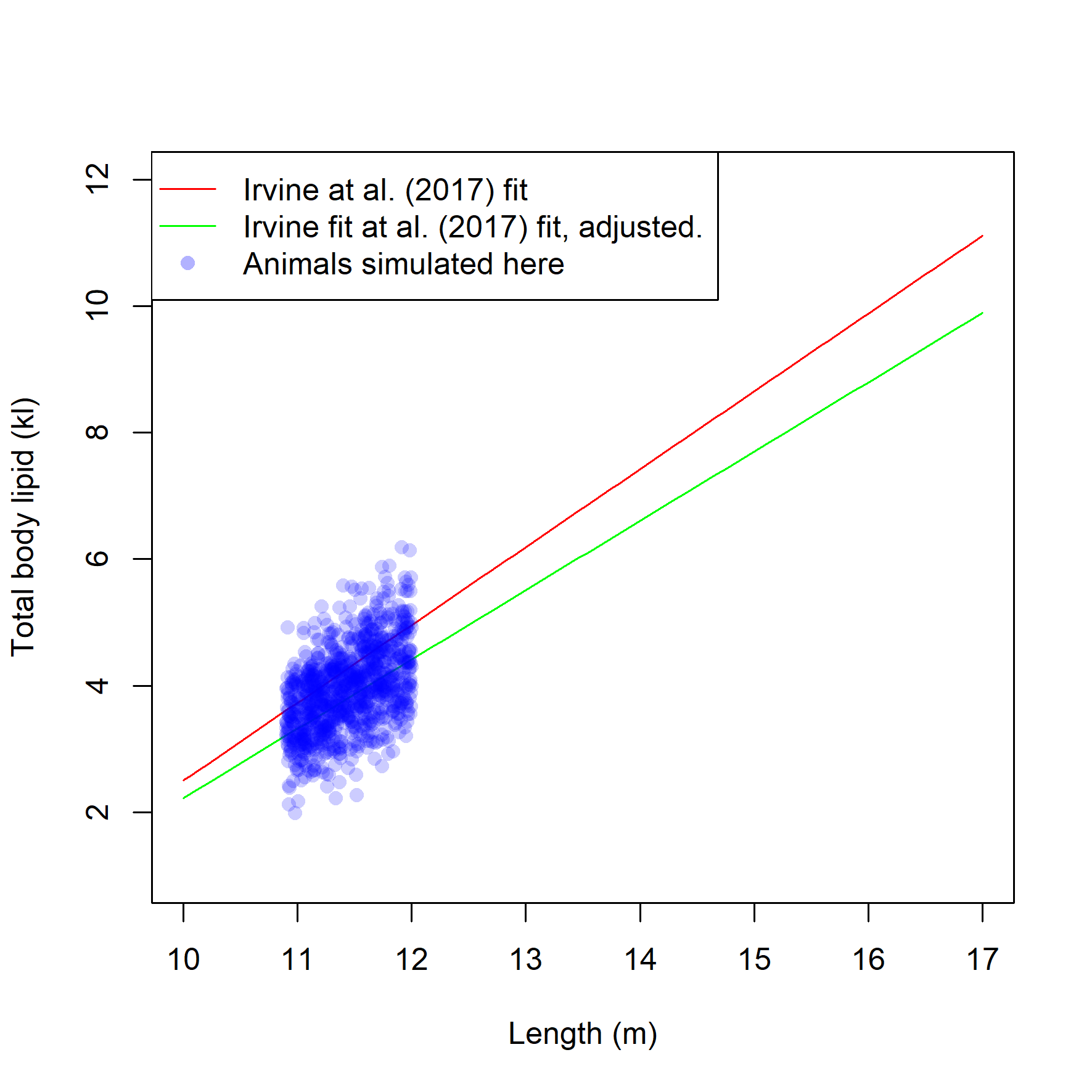


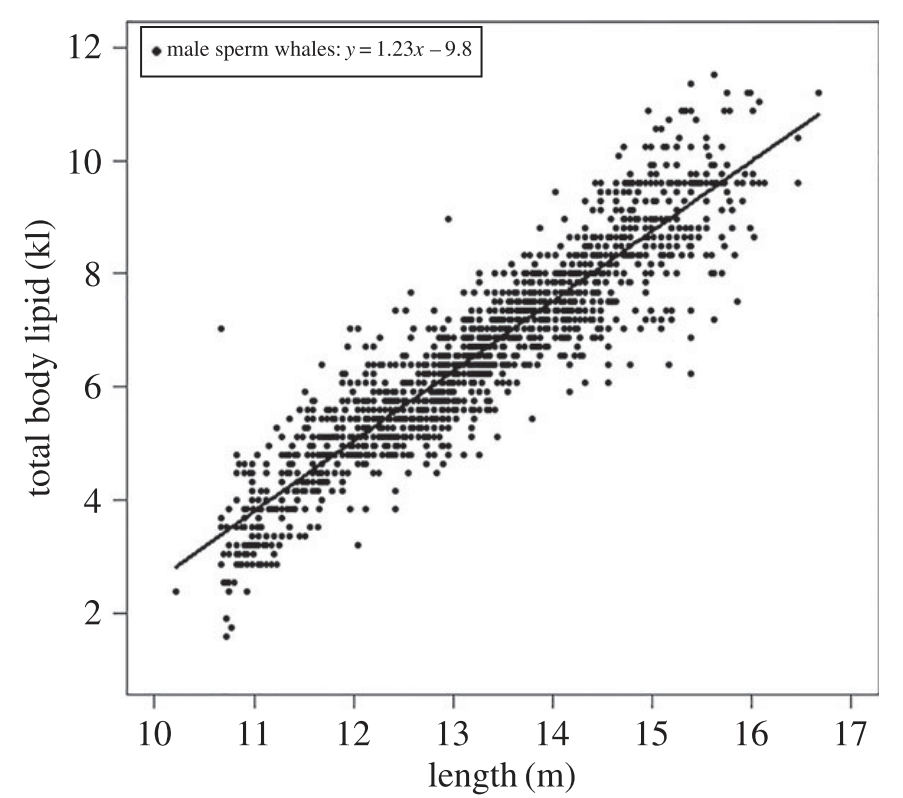


Figure S3: Comparison of simulated total body lipids with empirical measurements. The left panel (reproduced from Irvine et.al 2017) shows the raw data and fitted total yield to body length relationship for Australian sperm whale catch records. The right panel shows the same fitted relationship both with and without an 11% reduction to account for spermaceti yield, overlaid with the length and total blubber lipid values for the whales in our simulations.

**Literature cited**

Clarke R (1978) Structure and Proportions of the Spermaceti Organ in the Sperm Whale. *J Mar Biol Assoc United Kingdom* 58: 1–17. doi:10.1017/S0025315400024371

Dieterichs EEF (1906) A Practical Treatise on Friction, Lubrication, Fats and Oils, Including the Manufacture of Lubricating Oils, Leather Oils, Paint Oils, Solid Lubricants and Greases, Modes of Testing Oils, and the Application of Lubricants. HC Baird & Co., Philadelphia. p23

Irvine LG, Thums M, Hanson CE, McMahon CR, Hindell MA (2017) Quantifying the energy stores of capital breeding humpback whales and income breeding sperm whales using historical whaling records. *R Soc Open Sci* 4. doi:10.1098/rsos.160290

Lockyer C (1991) Body composition of the sperm whale, Physeter catodon , with special reference to the possible functions of fat depots. *J Mar Res Institute, Reykjavik* 12: 1–25.
